# Continuous Flat Pressing of MDF Quality Control Model Framework and Collaborative Programming Approach Based on Wood Fiber Hot Pressing Mechanism

**DOI:** 10.3389/fpls.2022.851219

**Published:** 2022-04-26

**Authors:** Yunlei Lv, Yaqiu Liu, Weipeng Jing

**Affiliations:** College of Information and Computer Engineering, Northeast Forestry University, Harbin, China

**Keywords:** wood fiber hot-pressing mechanism, MDF, sequence parameter control, conceptual digital twin modeling, optimization of agile forestry production, precision control

## Abstract

The increasing demand for forestry resources is driving the need for smarter systems capable of saving and protecting forests that can optimize agile forestry production. This study uses the continuous hot-pressing process of wooden medium-density fiberboard (MDF) to investigate the possibility of automatic quality control of the continuous flat pressing process. For this purpose, conceptual digital twin modeling for mechanism and sequence parameter control was conducted based on the cellular automata (CA) theory. A distributed coordination mode framework was constructed, and a craft control programming method was proposed for the quality control of MDF continuous flat pressing. Based on the MDF continuous flat press craft mechanism and control standards, a framework of five distributed flat press cooperative control mode elements for the cylinder array of the continuous panel system (CPS) was defined. To satisfy the distributed distance servo and pressure servo demands of the multi-stage hot pressing craft design, five kinds of synergy collaborative control modes of multiple rack groups were constructed using mode elements: For the four types of typical deviations in slab production, i.e., thickness, slope, depression, and bulge, a multi-zone mutual cooperative mode craft control sequence was programmed. According to the type and intensity of real-time deviation, the corresponding regulation sequence was applied. This effectively counteracts the deviation caused by the uncertainty interference due to the multi-field coupling effect in actual production. The application tests demonstrate that the adjustment and response time of the continuous flat press were greatly improved, and the quality superiority rate is controlled above 95%, thereby confirming the effectiveness of the control strategy.

## 1. Introduction

Currently, the global forest stock volume is limited to approximately 540 billion *m*^3^ and is declining every year. However, the wood-based panel industry continues to consume a significant amount of forest resources, with a global annual output greater than 400 million *m*^3^ (Linjun, 2021). To achieve the sustainable development of forest ecology and the effective protection of forest resources, it is imperative for the wood-based panel industry to make technological progress and optimize its crafting operations to reduce unnecessary production loss (Rongsheng, 2017).

Medium-density fiberboard (MDF) is a type of wood-based panel made of wood fiber or other plant fibers, treated with urea-formaldehyde resin or other applicable adhesives, and formed by hot pressing; its density is generally 500–880 kg / *m*^3^(Wanli, 2019). MDF has become the pillar industry of wood-based panel boards owing to its excellent material properties, stable performance, and wide applicability. It is a technology-intensive industry with the most complex craft and the highest degree of automation in the production of wood-based panels. Continuous hot pressing, especially continuous flat pressing technology, has become the central procedure in the large-scale production of MDF because of its features such as craft continuity, high productivity, excellent product quality, accurate shape control, and a wide variety of product types. Moreover, the process is easily adjustable, saves resources and energy, and has several comprehensive benefits. In addition, it consistently meets the ever-increasing demand for panel quality while reducing production loss by effectively utilizing resources. A continuous panel system (CPS) has been recognized as crucial equipment by the global market and has been subjected to continuous innovation and development (Xinqing, 2021). At present, the new-generation technology of continuous pressing CPS+ released by Dieffenbacher in 2015 in the field of international wood-based panels implements the German Industry 4.0 application and automation integration by modularized design. Pre-process production, raw material preparation, craft control, online equipment monitoring, diagnosis, and maintenance are integrated through digital twin technology and big data to make the panel production flow more intelligent, flexible, and efficient (Yaqiu et al., 2014).

The quality control of the plain slab comprises a novel cylinder arrangement of CPS+ continuous press. In the new design, the primary cylinder pressure acts directly on the plate, thereby making transverse pressure adjustment more flexible. Moreover, the parallel-gap design of the press limits the deformation between the frames; the thickness control zone of the plain slab is optimally designed; the transverse thickness tolerance can be controlled within a small variation range; and the quality of the slab surface and edge has been further improved. The optimized design of the control system keeps the gap consistent between the upper and lower plates in both states of the cylinder: pressured and unpressured (distance servo). In addition, it maintains the longitudinal slab pressure servo on the hot press zone of multiple cylinder frames and avoids the adverse effect of viscoelasticity on the thickness control of slab formation. Furthermore, the design enables the production of slabs with a smaller thickness tolerance and better surface quality, thereby saving material. In distributed control, in Shao et al. (2015), the primary craft parameters including the hot press plate gap, pressure, temperature, and press closure speed, were analyzed by studying the MDF production craft and its process. Following this, a multi-field coupling effect MDF process model was proposed based on the viscoelastic structure theory; the servomechanism and hydraulic loading system were analyzed, and a hydraulic distance servo control system model with pressure negative feedback was constructed by introducing the slab compressive stiffness and hydraulic stiffness coupling factors, research the mechanism of automatic shape control (AFC), consider the viscoelastic constraints of the slab, plan the pressure distribution and displacement strategy in the forming interval; optimize the design AFC process of the power unit execution mode of the high-efficiency viscoelastic. In Yang et al. (2019), based on the research on the distance tracking control problem with nonlinear interference, internal parameter perturbation, and external load disturbance, a mathematical model for the hydraulic system controlling the slab thickness was built. In Li et al. (2011), a multi-agent distributed event-triggered control method was proposed based on the leader-following network, in which the regulation goal enables the master–slave structural unit to achieve consistency control. The problem of the large-scale network with a large number of agents was solved by transforming the leader-following consensus (LFC) problem into one leader and two followers. In addition, the master–slave structural unit was constructed to achieve consistency control by transforming the LFC into a synchronization problem with one leader and one follower. It included establishing a single terminal displacement control system and static load pressure control model, deriving the state equation and the expression of the cooperative control law of the corresponding system, and designing the corresponding cooperative controller (Bao, 2006). Another significant method is the cellular automata (CA) theoretical method, which is used to study the overall behavior (emerging) of a system based on local interactions (local behavior). It has a high degree of flexibility and freedom to describe the local behavior of complex systems, and the cell has the characteristics of synchronization between state discretization, interaction, and dynamic evolution (Zhu et al., 2018).

It must, however, be noted that certain limitations exist in the abovementioned frontier research, as well as in actual production. In particular, the synergy mode of the distributed dynamic actuating unit and deviation quality control method of the continuous flat press control system suffer from a lack of craft mechanism analysis, systematic definition, and theoretical basis. Thus, the model adopted in this study aims to comprehensively consider the multi-field coupling effect of hot press forming and innovatively apply the state characteristics and evolution rules of the cylinder array cellular automaton. For this purpose, we conducted research on the conceptual digital twin modeling for the distributed control mode framework and quality control sequence parameter optimization programming of MDF CPS. We subsequently analyzed and constructed five control mode types and five mutual synergy mode frameworks, according to the slab-making craft with viscoelastic and consistency control of the dynamic plate surface. Finally, we programmed the synergistic control sequence with dual functions of distance servo and pressure servo for the longitudinal hot press zone of multiple cylinder frames, to satisfy the quality control requirements, including convenient adjustment and accurate slab formation.

## 2. Wood Fiber Hot-Pressing Mechanism and Related Studies

From the perspective of slab characteristics and mechanism, MDF slab is a type of medium with a dense gap, which has the functions of heat, mass, and momentum transfer during the hot-pressing process. Complex chemical reactions and phase changes can also occur in this medium (Linjun, 2021). The primary reasons for the above changes are the heat and mass transfer, specific heat, thermal and temperature conductivity, properties of the adhesives, etc., which are important indicators in the wood fiber hot-pressing process. During MDF hot pressing, the plate is in direct contact with the surface of the panel, and the heat is transferred to the surface layer, after which it gradually diffuses to the core layer of the slab. As the pressure increases, the heat, pressure, and water vapor inside the slab undergo a series of complex changes. Simultaneously, the body of the slab is softened, the adhesive is gradually cured by heat, and the slab is compressed. The density of the fluffy MDF is lower than that of solid wood prior to hot pressing and higher than it after the process. There are two primary methods of heat transfer: heat conduction and heat convection during hot pressing.


(1) $$\begin{array}{l} \tau_{1}=\frac{h^{2}}{\pi^{2}\alpha_{1}}ln\;\left(\frac{4}{\pi }\cdot \frac{t_{c}-t_{1}}{t_{c}-t_{0}}\right) \end{array}$$

where *t*_1_ is the temperature of the core layer of the panel in the first stage (°C), and α_1_ is the temperature conductivity coefficient in the direction perpendicular to the plane of the slab in the first stage (*cm*^2^/*s*). In the second water vaporization stage, most of the heat in the core layer acts on the vaporization of moisture, and the temperature remains constant. The time required for the vaporization stage is


(2) $$\begin{array}{l} \tau_{2}=\frac{rm_{w}}{q} \end{array}$$

where *q* is the heat provided to the inside of the panel for the unit time by the hot-pressing plate (*kJ*/*kg*), and *m*_*w*_ is the total mass of moisture inside the slab (kg). The third stage is a slow heating stage, which is basically the same as the first-stage heating method, except that the starting temperature is higher and the heating speed is slower. The required heating time is expressed in Equation (3):


(3) $$\begin{array}{l} \tau_{1}=\frac{h^{2}}{\pi^{2}\alpha_{2}}ln\;\left(\frac{4}{\pi }\cdot \frac{t_{c}-t_{2}}{t_{c}-t_{1}}\right) \end{array}$$

Where α_2_ is the temperature conductivity coefficient of the third stage perpendicular to the panel plane (*cm*^2^/*s*). Therefore, in the MDF hot pressing whole stage, the heat and mass transfer model [13] in the case where the temperature of slab core layer reaches within the required time τ is:


(4) $$\begin{array}{l} \tau =\tau_{1}+\tau_{2}+\tau_{3}=\frac{h^{2}}{\pi^{2}\alpha_{1}}\ln\left(\frac{4}{\pi }\cdot \frac{t_{c}-t_{1}}{t_{c}-t_{0}}\right)+\frac{rm_{w}}{q}\\ \;+\frac{h^{2}}{\pi^{2}\alpha_{1}}ln\;\left(\frac{4}{\pi }\cdot \frac{t_{c}-t_{2}}{t_{c}-t_{1}}\right) \end{array}$$

**Figure 1:**
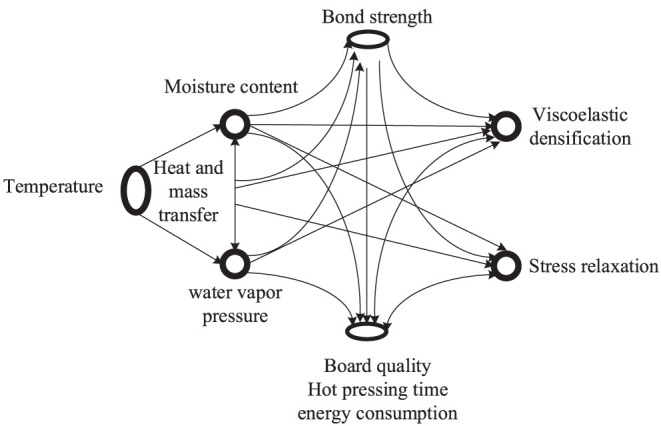
Multi-field coupling effect diagram.

Under the action of the temperature gradient, the distribution of temperature field and moisture content field inside the slab, such as pressure, temperature, moisture content, gas, and glue curing reaction, the ratio of moisture content gradient to the temperature gradient, and its influencing factors establish a multi-field coupling effect model as shown in Figure 1:


(5) $$\begin{array}{l} {\left(\rho c_{p}\right)}_{e}\frac{{\partial \langle T\rangle }^{r}}{\partial t}=\nabla \cdot \left(k_{e}\cdot \nabla {\langle T\rangle }^{r}\right)+\Phi_{r\rho_{r}}H_{R}R_{C} \end{array}$$

The hot-pressing plate is in direct contact with the surface of the MDF panel during the hot pressing process, and heat is transferred to the surface layer of the slab and then gradually diffuses to the core layer fibers. As the pressure rises, the heat, pressure, and water vapor inside the slab undergo a series of complex changes. Simultaneously, the fiber matrix is softened, and the adhesive is gradually cured by heat, causing the slab to be compressed to form.

**Figure 2:**
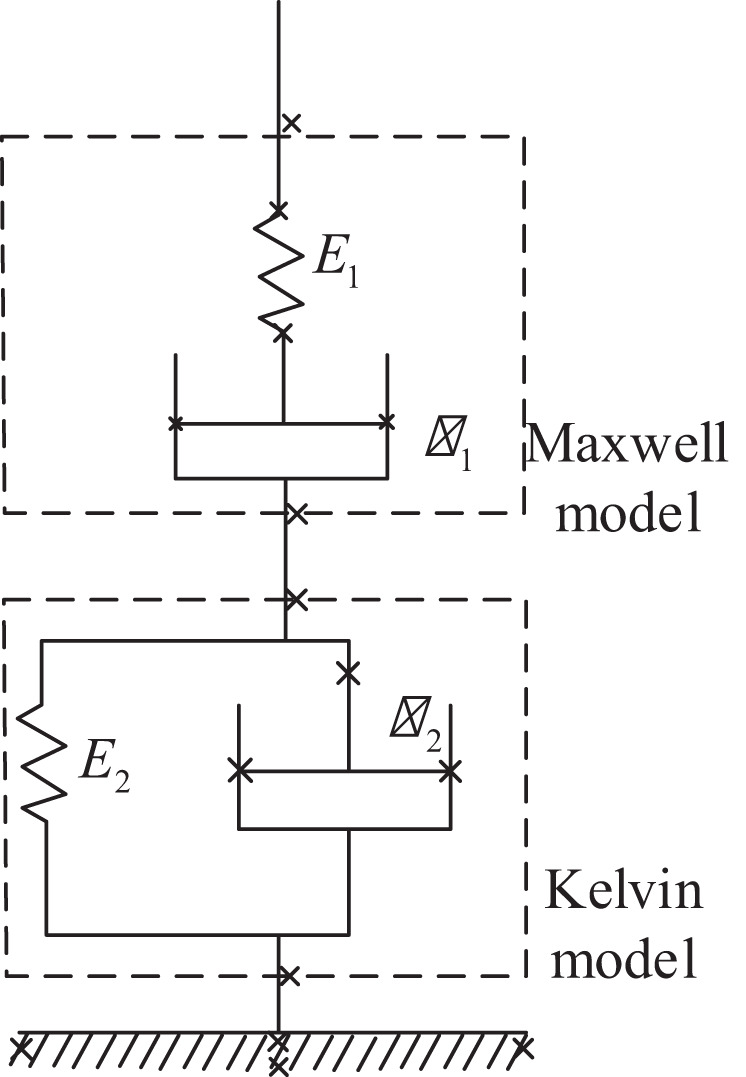
Burgers model de-viscoelastically of medium-density fiberboard (MDF) pressure.

Where ρ is the density, *T* is the temperature, *c*_*p*_ is the specific heat capacity, *R*_*C*_ and *H*_*R*_ are the curing rate and the curing heat per unit mass, respectively; and *k*_*r*_ and *k*_*f*_ are the heat transfer coefficient tensors, including heat conduction and dissipation effects. For the hot pressing process, in a time scale comparable to the process cycle, the temperature difference between the glue and the fiber is small, which is equivalent to the two being equal to 〈*T*〉^*r*^ = 〈*T*〉^*f*^; *k*_*e*_ = Φ_*r*_*k*_*r*_ + Φ_*f*_*k*_*f*_ is the effective heat transfer coefficient tensor; (ρ*c*_*p*_)_*e*_ = Φ_*r*_ (ρ*c*_*p*_)_*r*_ + Φ_*f*_ (ρ*c*_*p*_)_*f*_ is the effective heat capacity. Considering the influence of the inherent viscoelasticity of the slab on the quality control of the MDF, viscoelastic parameters were introduced to improve the robustness and stability of the system, and the output parameters of the controller are optimized, without considering viscoelasticity, using the Burgers model, which is a four-element model comprising the Maxwell basic model and the Kelvin basic model, as shown in Figure 2, the “ × ” in the figure represents the region where the viscoelasticity exists in the word model.

where *E*_1_ and *E*_2_ represent the spring stiffness of the elastic element in the Maxwell and Kelvin models, respectively, and η_1_ and η_2_ represent the viscosity coefficient in the Maxwell and Kelvin models, respectively; for the elastic element, the relationship between stress and strain is σ = *E* × ε. In Maxwell's basic model, the viscous element is a simple series relationship, that is, ε = ε_1_ + ε_2_,$\overset{.}{\varepsilon }=\varepsilon_{1}+\overset{.}{\varepsilon_{2}}$; *E* is the elastic stiffness, and the constitutive model equation is constructed by Maxwell and Kelvin in Equation (6).


(6) $$\begin{array}{l} \overset{.}{\varepsilon_{1}}=\frac{1}{E_{1}}\overset{.}{\sigma }+\frac{1}{\eta_{1}}\sigma \end{array}$$

The constructed burgers model of slab is Equation (7):


(7) $$\begin{array}{l} \frac{\eta_{1}E_{1}+\eta_{1}E_{2}+\eta_{2}E_{1}}{E_{1}E_{2}}\overset{.}{\sigma }+\frac{\eta_{1}\eta_{2}}{E_{1}E_{2}}\overset{.}{\sigma }=\eta_{1}\overset{.}{\varepsilon }+\frac{\eta_{1}\eta_{2}}{E_{2}}\overset{.}{\varepsilon } \end{array}$$

Design under the condition that the slab has viscoelasticity, model identification, and devised a synergistic model framework and synergistic planning method through the effect of synergy in the viscoelastic situation through a master–slave structure approach.

**Figure 3:**
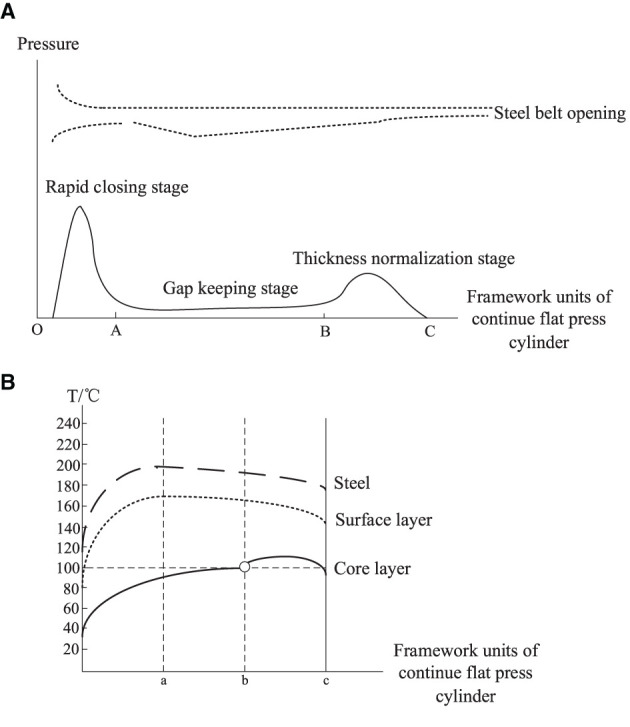
Trend chart of baseline craft curve of hot-pressing mechanism.

From the perspective of craft mechanism, MDF continuous flat press is divided into 3 zones: rapid closing stage, gap keeping stage, and thickness normalization stage (Shun Wu, 2008), as shown in Figure 3, the main parameters of the craft baseline are based on the pressure, opening, and temperature during the hot pressing process. The opening and closing degree of the steel strip corresponds to the three stages of the process. The goal of craft control is to keep the gap setting curve consistent with the actual curve, that the gap between upper and lower plates has not the same setting curves in different zones according to panel materials, thickness, and density requirements.

(1) In the rapid closing stage, the gap between the plates is gradually reduced, so that the panel can be pressed to a certain thickness in a short time, but the setting of the change slope is to satisfy the pre-pressing panel structure is complete and a certain density of solidified layer is formed on the surface. (2) In the gap keeping stage, due to a large amount of water vapor generated, the gap of the flat press must be opened slowly to a certain extent to facilitate the release of heat vapor mass and does not affect the subsequent processing and final physical properties of the panel, that change curve of the press gap opening is correspondingly set according to the production of different panels. (3) In the thickness normalization stage, the thickness of the panel is greatly different from the set thickness *via* the gap keeping stage. It is necessary to adjust the gap opening near the exit of the flat press to regularize the thickness of the panel. Generally, considering the rebound characteristics of the panel, the setting value of the gap of the thickness normalization stage should be slightly smaller than the setting value of the panel thickness.

**Figure 4:**
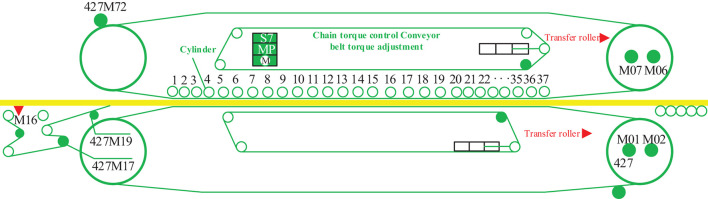
Legend of the continuous panel system (CPS).

As an example, the new generation of CPS+ (Zhou, 2021) with 37 group frames structure is shown in Figure 4.

In Figure 4, frame numbers 1–37 are independent dynamic actuating units, or units in groups of two to three rows, which can be controlled in different zones according to the different process stages of the flat pressing craft. The M01, M02, M07, M06, M16, M17, M19, M72 are motor numbers. The continuous flat press pressure primarily comprises the reaction force provided by the flat press to overcome the rebound of the panel. The pressure promotes the exhaust of gas from the inside panel, which accelerates the heat transfer, increases the touching and interweaving between fibers, and makes the product dense to achieve the required thickness (Zhu et al., 2021). Therefore, the continuous flat press is divided into three zones for pressure control: a high-degree pressure zone, a low-degree pressure zone, and a re-pressure zone. Owing to the variation pattern of the internal and external environments (pressure, temperature, humidity, gas, etc.) of the panel in the continuous flat pressing process, MDF acquires its own viscoelastic properties and a complex structure. It is required that the MDF continuous flat press process has two dynamic output modes: distance servo and pressure servo, and the control model is analyzed in two cases.

**(1) The distance servo model with output as piston displacement of the cylinder**

By establishing a single-terminal and multi-terminal coordination structure in the hydraulic cylinder array, a coordination mechanism for the automatic correction of continuous slab thickness was proposed in Chao (2001). Thickness control is transformed into the process of compensation of the panel thickness by the actuating unit of the press cylinder, where the piston reset displacement of the hydraulic cylinder is vector summed with the given displacement value so that the piston outputs the desired displacement. That is, the dynamic system is a hydraulic oil discharge control system (Li, 2016; Zhu et al., 2019).


(8) $$\begin{array}{l} D=D_{r}+D_{f}=\frac{\frac{k_{q}}{A}X_{v}-\frac{k_{ce}}{A^{2}}\;\cdot \;Z_{f}\left(1+\frac{s}{\omega_{1}}\right)F}{\omega_{2}\left(\frac{s}{\omega_{r}}\;\cdot \;{\overset{.}{Z}}_{f}+1\right)\left(\frac{s^{2}}{\omega_{0}^{2}}\;\cdot \;{\overset{..}{Z}}_{f}+\frac{2\delta_{0}s}{\omega_{0}}+1\right)} \end{array}$$

(9) $$\begin{array}{l} \{\begin{array}{l} D_{r}=\frac{k_{q}X_{v}}{AF}\;\cdot \;{\overset{.}{Z}}_{f}\;\\ D_{f}=-\frac{k_{ce}}{A^{2}}\left(1+\frac{s}{\omega_{1}}\right)\;\cdot \;{\overset{..}{Z}}_{f} \end{array} \end{array}$$

where *F* is the external load force, *D* is the piston displacement, *D*_*r*_,*D*_*f*_ are the piston reset displacement and the given displacement, *A* is the effective area of the piston, *x*_*v*_ is the displacement of the four-way valve spool, and *k*_*ce*_ is the servo valve gain. Let ω_1_ represent the ratio of hydraulic spring stiffness to hydraulic damping, ω_2_ represent the ratio of load stiffness to damping system, and ω_*r*_ represent the ratio of hydraulic spring stiffness to the stiffness and damping coefficient when coupled with the load spring.

Let *x*_1_ = *Z*_*f*_ $x_{2}={\overset{.}{Z}}_{f}$ $x_{3}={\overset{..}{Z}}_{f}$, lead to the displacement control system states equation


(10) $$\begin{array}{l} \{\begin{array}{l} \overset{.}{x_{1}}=x_{2}\;\\ \overset{.}{x_{2}}=x_{3}\;\\ \overset{.}{x_{3}}=f_{D}\left(x_{1},x_{2},x_{3}\right)+g_{D}\left(x_{1},x_{2},x_{3}\right)\;u \end{array} \end{array}$$

In Equation (10), *x*_1_, *x*_2_, *x*_3_ is the transfer mode from the analysis [10]. Substituting Equation (10) into Equations (8) and (9), the system output equation for distance can be obtained as follows:


(11) $$\begin{array}{l} \{\begin{array}{c} f_{D}\;(x_{1},x_{2},x_{3})=-\left(2\varsigma_{0}\omega_{0}+\omega_{r}\right)\;x_{3}\\ -\left(\omega_{0}^{2}+2\varsigma_{0}\omega_{r}\omega_{0}\right)\;x_{2}-\omega_{r}\omega_{0}^{2}x_{1}\\ g_{D}\;(x_{1},x_{2},x_{3})=\frac{\omega_{r}\omega_{0}^{2}k_{ce}}{\omega_{2}}x_{1}^{-1} \end{array} \end{array}$$

In Equation (11), *f*_*D*_ and *g*_*D*_ are the displacement outputs in the state function.

**(2) The pressure servo model with output as cylinder pressure on the plate**

In the process of panel thickness correction, the non-fixed thickness cylinders are responsible for maintaining the required pressure during panel formation; i.e., the dynamic system is a static load pressure control system. In such a system with viscoelastic load (He et al., 2017), Equation (5) represents a static load pressure output system based on the working principle of the four-way valve (Lv et al., 2020). The state function equation of the static load pressure control system is expressed in Equation (12):


(12) $$\begin{array}{l} \{\begin{array}{c} f_{P}\;(x_{1},x_{2},x_{3})=-\left(2\varsigma_{0}\omega_{0}+\omega_{r}\right)\;x_{3}\\ -\left(\omega_{0}^{2}+2\varsigma_{0}\omega_{r}\omega_{0}\right)\;x_{2}-\omega_{r}\omega_{0}^{2}x_{1}\\ g_{P}\;(x_{1},x_{2},x_{3})=\omega_{r}\omega_{0}^{2}k_{sv}x_{2} \end{array} \end{array}$$

In Equation (12), *f*_*P*_ and *g*_*P*_ are the displacement outputs in the state of the state function.

To achieve accurate control of panel forming in MDF production, a press control system must be capable of flexible and effective regulation of the quality control of panel thickness and normalization. The master–slave structure mode and distributed event-triggered control method are applied to the craft cooperative control of cylinder arrays of the CPS for the typically generated quality deviations: panel thickness, slope, depression, and bulge. Through the above method, the framework of the distributed cooperation control mode and the craft control sequence programming method were researched.

## 3. Framework Construction of MDF Continuous Flat Pressing Control Mode

### 3.1. Description of Distributed Cylinder Array

**Figure 5:**
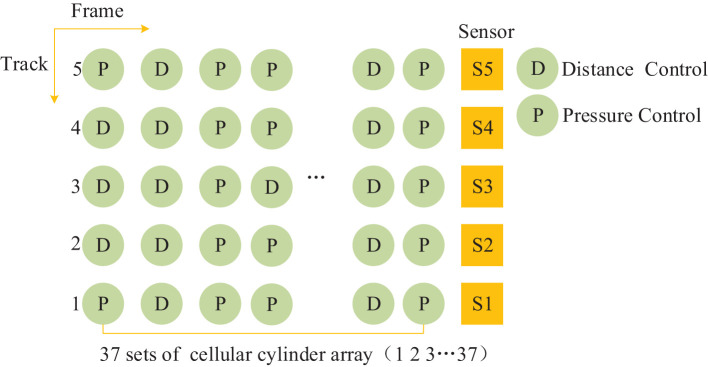
The cylinders array of distribution MDF continuous flat press.

The MDF continuous flat pressing cylinders were arranged on dynamic action surfaces in the frame. A continuous flat pressing craft was used to construct the control mode frame in collaborative modes of 37*5 as an example. The distribution of cellular cylinder arrays constructed using digital twin mapping between digital twin mapping technology and fiber forming is shown in Figure 5.

Figure 5 is the distribution is constructed with CPS+ as the object. It is divided into 37 groups of pressure cellular cylinders, D and P indicate the types of sensors in Figure 4. The mode is not fixed and unique, and the situation in the figure is a basic example. Analyze from the frame (transverse) and pass (longitudinal) directions to realize distance servo and pressure servo control.

### 3.2. Self-Coordinated Working Mode Framework Definition of Cylinders

**Figure 6:**
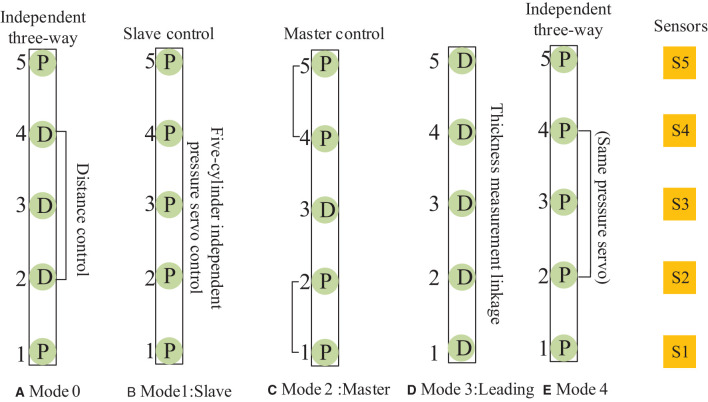
The framework of the control mode of the continuous flat cylinder array.

According to the working mode, the cellular cylinder group of MDF continuous flat pressing was defined, and the control mode analysis was constructed as shown in Table 1. The frame of the MDF continuous flat pressing control mode is shown in Figure 6. Digital twin technology is used to form a digital twin mapping between the framework of the collaborative mode and the fiber-forming mechanism.

**Table 1:** Analysis table of medium-density fiberboard (MDF) continuous flat pressing control mode.

**Cylinders working mode**	**Cylinder group mode sensor sensing type**	**Self-coordination**	**Mutual coordination**
Mode 0 (Independent three-way)	Pressure(Distance,Distance,Distance) Pressure	Yes	Yes
Mode 1 (Slave)	Pressure,Pressure,Pressure,Pressure,Pressure	Yes	Yes
Mode 2 (Master)	(Pressure,Pressure)Distance(Pressure,Pressure)	Yes	Yes
Mode 3 (Leading)	Distance,Distance,Distance,Distance,Distance	Yes	Yes
Mode 4 (Independent three-way)	Pressure(Pressure,Pressure,Pressure)Pressure	Yes	Yes

The control mode 0, shown in Figure 6A, is the cylinder position-pressure self-coordinated control of a single frame, i.e., the positions of cylinders 2, 3, and 4 are controlled independently, and both sides are controlled by a pressure follower (cylinders 1 and 5 are pressure servo) control. Control mode 1, shown in Figure 6B, is the slave control mode, i.e., the independent pressure control of five cylinders in the same frame. Control mode 2, shown in Figure 6C, is the master control mode, i.e., the middle cylinder 3 is the primary position control, and cylinders 1, 2 and cylinders 4, 5 are pressure following slave control. Control mode 3, shown in Figure 6D, is the leading mode, which is the independent control of the position of the five cylinders, i.e., the cylinders of the front and rear frames follow the corresponding cylinders of the main frame to achieve thickness linkage. Control mode 4, shown in Figure 6E, is the cylinder pressure self-coordinated control of a single frame, i.e., the pressures of cylinders 2, 3, and 4 are controlled independently, and the two sides (1, 5) are separated by pressure following control.

### 3.3. Mutual Coordination Control Method Between Cylinder Groups in Different Frames

**Figure 7:**
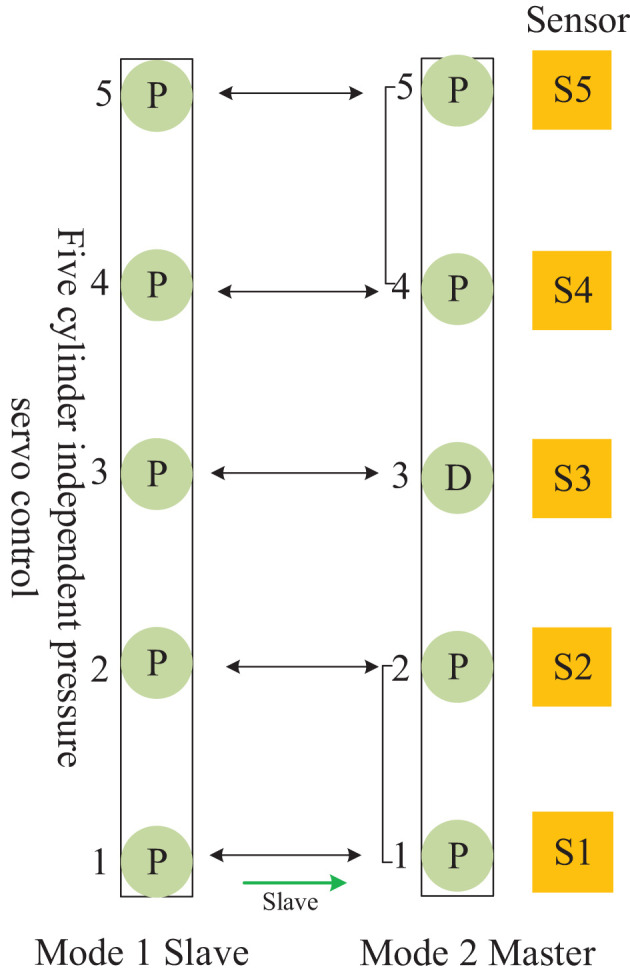
Distributed cooperative control mode of continuous flat pressing cylinder array Mutual cooperation A.

**Figure 8:**
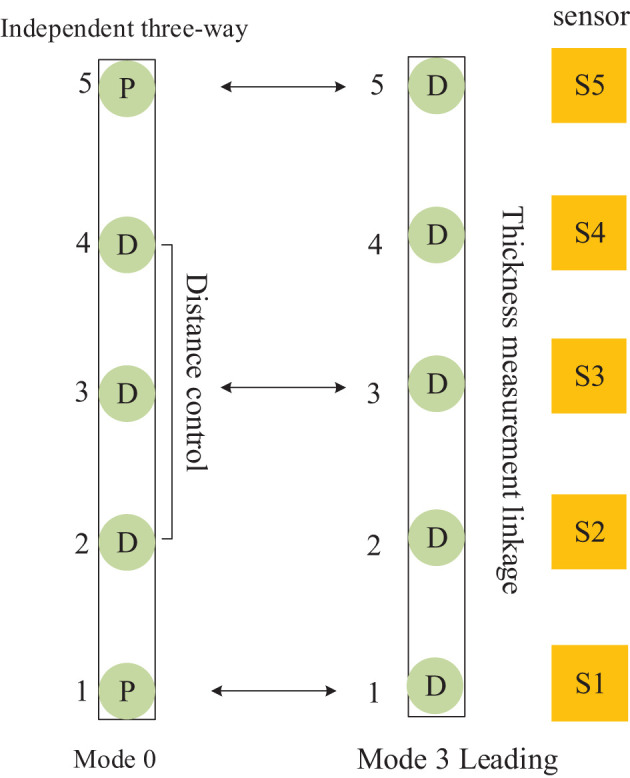
Distributed cooperative control mode of continuous flat cylinder array Mutual cooperation B.

**Figure 9:**
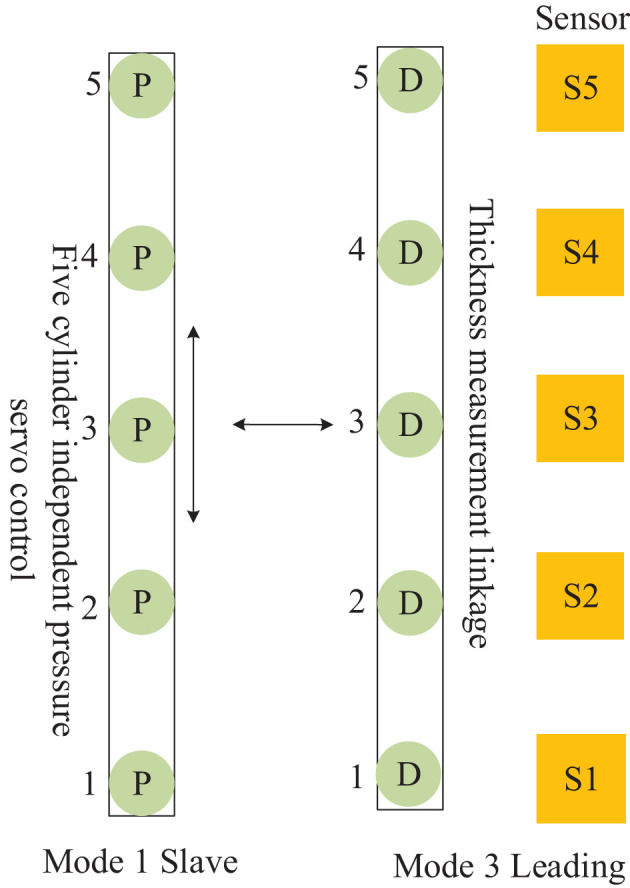
Distributed cooperative control mode of continuous flat cylinder array Mutual cooperation C.

**Figure 10:**
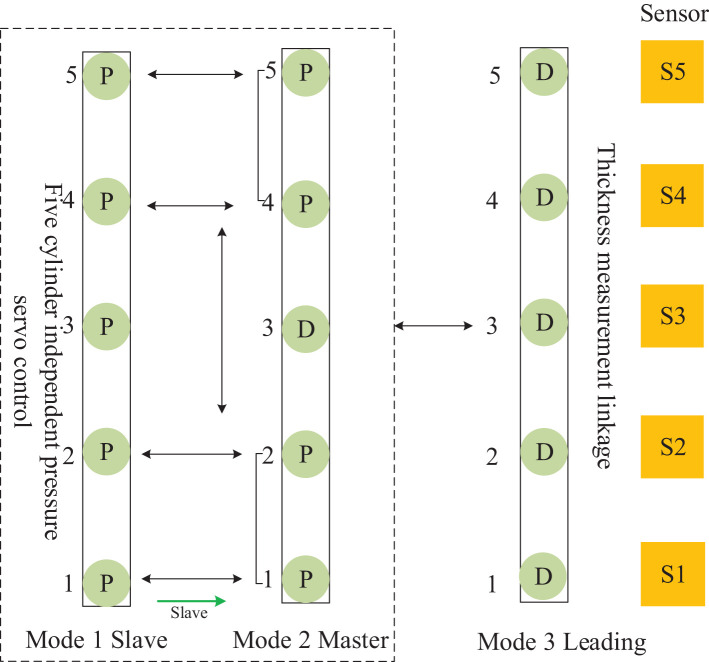
Distributed cooperative control mode of continuous flat cylinder array Mutual cooperation D.

**Figure 11:**
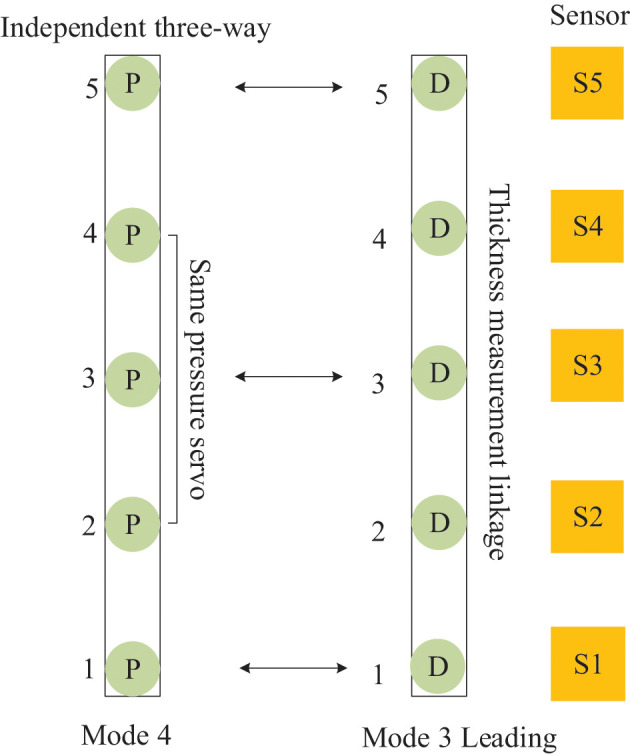
Distributed cooperative control mode of continuous flat cylinder array Mutual cooperation E.

Considering that the cylinders of the CPS on the continuous flat pressing plate are arranged in an array, the dynamic surface control of the plate can be transferred to the coordinated control of the hydraulic cylinder array. The mutual cooperation mode framework of the frame cellular group was analyzed, as shown in Table 2, and the mutual cooperation control formation type was defined, as shown in Figures 7–**11**.

**Table 2:** Frameworks analysis of mutual frame cooperation mode.

**Mutual cooperation model**	**Formation type**	**Other same model**
Mutual cooperation mode A	1,2	or 2, 1 or 1, 2, 1
Mutual cooperation mode B	0,3	or 3,0
Mutual cooperation mode C	1,3	or 3,1 or 1,3,1
Mutual cooperation mode D	1,2,3	or 3, 2, 1 or 1, 2, 3, 2, 1
Mutual cooperation mode E	1,4	or 4, 1 or 1, 4, 1

The mutual cooperation mode A defined in Figure 7, as the master–slave type, comprises both frames of the cellular cylinders working in modes 1 and 2, and the slave frame cooperates with the master frame to complete the position pressure follow-up control.

The mutual cooperation mode B defined in Figure 8, comprises both frames of cellular cylinders working in modes 0 and 3, and the frame of cylinders working in mode 0 cooperates with the leading frame to complete the position follow-up control.

The mutual cooperation mode C defined in Figure 9, as the Leading-Slave type, comprises both frames of cellular cylinders working in modes 1 and 3, and the slave frame cooperates with the leading frame to complete the position pressure follow-up control.

The mutual cooperation mode D defined in Figure 10, as the Leading-Master type, comprises both frames of cellular cylinders working in modes 3 and 2. Here, in contrast to the other modes, the frames of cylinders working in mode 2 contain a master-slave sub-collaborative mode group, which cooperates with the leading frame to complete the position pressure follow-up control.

The mutual cooperation mode E defined in Figure 11, comprises both frames of cylinders working in modes 4 and 3, and the frame of cellular cylinders working in mode 4 cooperates with the leading frame to complete the position follow-up control. According to the mutual cooperation mode of the master–slave structure and the working principle of the four-way valve of the hydraulic system, a dynamic equation is designed in the master actuator *M*, and identical state parameters are received equally on the slave actuator S and master actuator *M* of the master–slave structure. Then, the system generates output under the condition of *M* restriction on S (He et al., 2018; FangFei Cao, 2021). For the control mode of the MDF continuous flat pressing process, the distance-pressure coordination mode of the cylinders is expressed as


(13) $$\begin{array}{l} M\;:\;\{\begin{array}{l} \overset{.}{x_{M}}\left(t\right)=P_{L}=\frac{k_{q}}{k_{ce}}x_{3}\left(t\right)+\frac{2k_{q}\zeta_{m}}{k_{ce}\omega_{m}}x_{3}\left(t\right)+\frac{k_{q}}{k_{ce}\omega_{m}^{2}}\\ \;z_{M}\left(t\right)=\;-\frac{\eta_{D}+Tf_{u\text{\_}D}\left(x_{p}\right)}{Tg_{u_{D}}\left(x_{p}\right)}x_{3}\left(t\right)\; \end{array} \end{array}$$

(14) $$\begin{array}{l} S\;:\;\{\begin{array}{l} \overset{.}{x_{S}}\left(t\right)=\frac{k_{q}}{k_{ce}}-\omega_{0}^{2}-2\zeta_{0}\omega_{r}\omega_{0}\left(t\right)\\ z_{S}\left(t\right)=\frac{2k_{q}\zeta_{m}}{k_{ce}\omega_{m}}-2\zeta_{0}\omega_{r}\omega_{2}\left(t\right)\; \end{array} \end{array}$$

The linkage between the cylinder groups is formed through the state equation of the synergistic effect of *x*_*M*_ and *z*_*M*_ between the MDF continuous flat pressure cylinders *z*_*S*_ and *z*_*S*_, forming a master–slave structure.

Where *P*_*L*_ is the hydraulic driving force per unit area, *k*_*q*_ is the flow gain of the four-way valve, ζ_0_ is the flow-pressure coefficient of the four-way valve, and ω_*m*_ is the ratio of the mass of the piston to that of the damping system.

Where *x*_*M*_ the pressure change in this state is equivalent to *P*_*L*_, where *x*_2_, *x*_3_ are the representation methods of position-pressure coordinated control in the 2 and 3 states, and *z*_*M*_ represents the information of the control amount *u*_*D*_ in the control of the master–slave structure. Changes with time *t*, at this time the state equation of the continuous flat pressing process control of MDF changes with time t.

Let $\overset{.}{\eta_{D}=D}$, $\overset{.}{D}$ denote the state of position control, obtained


(15) $$\begin{array}{l} \eta_{D}=\overset{.}{D}=\left(\frac{X_{v}}{AF}-\frac{k_{ce}}{A^{2}}\right)x_{2}-\frac{k_{ce}}{A^{2}\omega_{1}}x_{3} \end{array}$$

Incorporate Equation (15) into Equation (8) distance state function:


(16) $$\begin{array}{l} \{\begin{array}{c} f_{u\text{\_}D}\left(x_{D}\right)=-\frac{k_{ce}}{A^{2}}\left(1-2\zeta_{0}\omega_{0}-\omega_{r}\right)\;x_{3}\\ -\left(\omega_{0}^{2}+2\zeta_{0}\omega_{r}\omega_{0}\right)\;x_{2}-\omega_{r}\omega_{0}^{2}x_{1}\\ g_{u\text{\_}D}\left(x_{D}\right)=\frac{k_{q}k_{sv}}{AF}x_{3}-\frac{k_{ce}\omega_{r}\omega_{0}^{2}k_{sv}}{A^{2}\omega_{1}\omega_{1}\omega_{t}}\; \end{array} \end{array}$$

*u*_*D* represents the control amount information in the distance state, and the two functions *f*_*u*_*D*_(*x*_*D*_)and *g*_*u*_*D*_(*x*_*D*_)are obtained from the control amount information, as shown in Equation (16). For the pressure cylinder coordination control of the MDF continuous flat pressing process, the pressure-pressure coordination mode of the cylinders is expressed as


(17) $$\begin{array}{l} M\;:\;\{\begin{array}{l} \overset{.}{x_{M}}\;(t)=-\frac{\eta_{p}+Tf_{u\text{\_}p}\left(x_{p}\right)}{Tg_{u_{p}}\left(x_{p}\right)}x_{3}\;(t)\;\\ z_{M}\;(t)=-\frac{\eta_{D}+Tf_{u\text{\_}D}\left(x_{p}\right)}{Tg_{u_{D}}\left(x_{p}\right)}x_{2}\;(t) \end{array} \end{array}$$

(18) $$\begin{array}{l} S\;:\;\{\begin{array}{l} \overset{.}{x_{s}}\left(t\right)=P_{L}=\frac{k_{q}}{k_{ce}}x_{2}\left(t\right)+\frac{2k_{q}\zeta_{m}}{k_{ce}\omega_{m}}x_{3}\left(t\right)+\frac{k_{q}}{k_{ce}\omega_{m}^{2}}\;\\ z_{s}\left(t\right)=\;-\frac{\eta_{D}+Tf_{u_{D}}\left(x_{p}\right)}{Tg_{u_{D}}\left(x_{p}\right)}\overset{.}{x_{2}}\;\left(t\right)\; \end{array} \end{array}$$

where *T* represents the convergence time for the system to reach the steady state in Equations (17) and (18), *u* represents the control value, and η_*D*_ represents the rate of change of the system distance output, which is also a variable function of the system state, where *x*_*M*_ is expressed as the pressure output state equation of the master control, *x*_*S*_ is equivalent to the pressure change in this state, and *x*_2_ and *x*_3_ are the expression methods of pressure-pressure coordinated control in 2 and 3 states, respectively. *Z*_*M*_ and *Z*_*S*_ represent the information of the control value *u*_*D* in the master–slave structure control, which changes with time *t*, which is the state equation of the current MDF continuous flat pressing process control. For the static load pressure control system, the control value is represented by *u*_*p*_, and η_*P*_ represents the rate of change of the system pressure output, which is also a variable function of the system state.


(19) $$\begin{array}{l} u_{p}=-\frac{\eta_{p}+Tf_{u\text{\_}p}\;(x_{p})}{Tg_{u\text{\_}p}\;(x_{p})} \end{array}$$

Let $\eta_{p}={\overset{.}{P}}_{L}$, then:


(20) $$\begin{array}{l} \eta_{p}={\overset{.}{P}}_{L}=\frac{k_{q}}{k_{ce}}x_{1}+\frac{2k_{q}\zeta_{m}}{k_{ce}\omega_{m}}x_{2}+\frac{k_{q}}{k_{ce}\omega_{m}^{2}}{\overset{.}{x}}_{3} \end{array}$$

(21) $$\begin{array}{l} M\;:\;\{\begin{array}{c} {\overset{.}{x}}_{u\text{\_}p\left(M\right)}\;(t)=\left(2\zeta_{0}\omega_{0}+\omega_{r}\right)\\ -\left(\left(\frac{k_{q}}{k_{ce}\omega_{m}^{2}}+2\zeta_{0}\omega_{r}\omega_{0}\right)+\omega_{r}\omega_{0}^{2}\right)-\omega_{r}\omega_{0}^{2}x_{1}\;(t)\\ z_{u\text{\_}p\left(M\right)}\;(t)=a_{2}\frac{2k_{q}\zeta_{m}}{k_{ce}\omega_{m}}x_{2}\;(t) \end{array} \end{array}$$

(22) $$\begin{array}{l} S\;:\;\{\begin{array}{l} {\overset{.}{x}}_{u\text{\_}P\left(S\right)}\;(t)=\omega_{r}\omega_{0}^{2}x_{1}\;(t)\;\\ z_{u\text{\_}P\left(S\right)}\;(t)=a_{2}\omega_{r}\omega_{0}^{2}k_{sv}x_{2}\;(t) \end{array} \end{array}$$

By combining the *x*_*u*_*P*(*M*)_, *x*_*u*_*P*(*S*)_ of the master-slave structure cooperative frames with ω_*r*_,$\omega_{0}^{2}$, and *x*_1_(*t*) in the four-way valve hydraulic control, a pressure coordination method between cylinders of different frames in the continuous flat pressing process of the MDF is formed, as shown in Equations (21) and (22). To regulate the craft quality control necessitated by the different types of panel deviation, the CPS multi-frame distributed coordinated control of distance-distance or distance-pressure is implemented between the cylinder zones of frame groups by the leading–follower scheme. This implementation is based on the single-frame cellular cylinder self-coordination of the master-slave structure. The system state equation for this is expressed as follows:


(23) $$\begin{array}{l} L\;:\;\overset{.}{x_{L}}\;(t)=a_{2}\omega_{r}\omega_{0}^{2}k_{sv}\overset{.}{x_{2}}\;(t) \end{array}$$

(24) $$\begin{array}{l} F\;:\;\overset{.}{x_{F}}\;(t)=\frac{k_{q}}{k_{ce}}x_{2}\;(t)+\frac{2k_{q}\zeta_{m}}{k_{ce}\omega_{m}}x_{3}\;(t)+\frac{k_{q}}{k_{ce}\omega_{m}^{2}}\overset{.}{x_{3}}\;(t)+\frac{4\beta_{e}C_{tc}k}{\omega V_{t}} \end{array}$$

The method is applied in a collaborative way in the continuous flattening process of MDF to make deviation corrections to achieve precise slab forming.

In the case of mutual cooperation between the controller of distance and pressure, both actuators output cooperative pressure through the collective effect to rectify deviation. The collaborative approach between the cylinder frames is configured through the mutual collaborative modes A–E, expressed in Equations (23) and (24), which then achieves the deviation quality control of the distributed coordination of the MDF in the continuous flat pressing process. The mutual cooperation modes A–E adopt the follow-up control method by distance-distance and distance-pressure follow-up on the master-slave system, in which the control strategy adopts the expert rule method.

According to the MDF production craft standard, combined with the CPS and on-site production experience, an expert regulation strategy was formulated, which will be used for real-time adaptive adjustment during press equipment operation to maintain product quality. In Tables 3, 4, the craft standard and deviation regulation collaborative rules are illustrated, taking the production of MDF with a thickness of 5.5 and 18.5 mm as examples.

**Table 3:** Collaborative rule table for 5.5 and 18.5 mm slab (distance-pressure).

**State**	**Position (D)**	**Pressure cylinder group pressure follow-up sequence**				
Craft standard	5.5mm	190 bar	200 bar	190 bar	190 bar	190 bar
Thickness deviation(bar)	+1%	+1%	+10%	+1%	+1%	+1%
Thickness deviation(+10)	+2%	+25%	+30%	+25%	+25%	+25%
Thickness deviation(+15)	+3%	+35%	+40%	+35%	+35%	+35%
Thickness deviation(+20)	+4%	+40%	+50%	+40%	+40%	+40%
Thickness deviation(+25)	+5%	+20%	+25%	+20%	+20%	+20%
Craft standard	18.5mm	170 bar	180 bar	190 bar	200 bar	180 bar
Thickness deviation(-10)	−2%	−15%	−9%	−15%	−15%	−15%
Thickness deviation(-15)	−3%	−25%	−20%	−25%	−25%	−25%
Thickness deviation(-20)	−4%	−35%	−30%	−35%	−35%	−35%
Thickness deviation(-25)	−5%	−45%	−40%	−45%	−45%	−45%

**Table 4:** Collaborative rule table for 5.5 and 18.5mm slab (distance-distance).

**State**	**Position (D)**	**Pressure cylinder group pressure follow-up sequence**				
Craft standard	5.5 mm	5.5 mm	5.5 mm	5.5 mm	5.5 mm	5.5 mm
Thickness deviation(+10)	+1%	0%	−2%	0%	0%	0%
Thickness deviation(+15)	+2%	0%	−2%	−4%	−2%	0%
Thickness deviation(+20)	+3%	0%	+4%	+4%	+5%	+5%
Thickness deviation(+25)	+4%	−2%	0%	+2%	+1%	−2%
Craft standard	18.5 mm	18.5 mm	18.5 mm	18.5 mm	18.5 mm	18.5 mm
Thickness deviation(-10)	+1%	0%	−2%	0%	0%	0%
Thickness deviation(-15)	−1%	+3%	−4%	−4%	0%	+3%
Thickness deviation(-20)	−2%	−1%	−1%	−1%	0%	0%
Thickness deviation(-25)	−3%	+2%	0%	0%	+2%	0%

In Table 3, the working modes of front 1, front 2, rear 1, rear 2, and rear 3 frames are respectively centered on mode 3 (Leading), and other frame pressure cylinders take positions according to the corresponding numbered distance-pressure follow-up control.

In Table 4, the working modes of front 1, front 2, rear 1, rear 2, and rear 3 frames are centered on mode 3 (Leading), and the other frame pressure cylinders take positions according to the corresponding numbered distance-distance follow-up control. According to the abovementioned craft control mode framework, the coordination mode between cylinder arrays of 37 cellular frames and the control coordination sequence programming of correction control in the case of deviations were analyzed.

### 3.4. Craft Control Sequence Parameter Programming for Accurate Panel Forming

**Figure 12:**
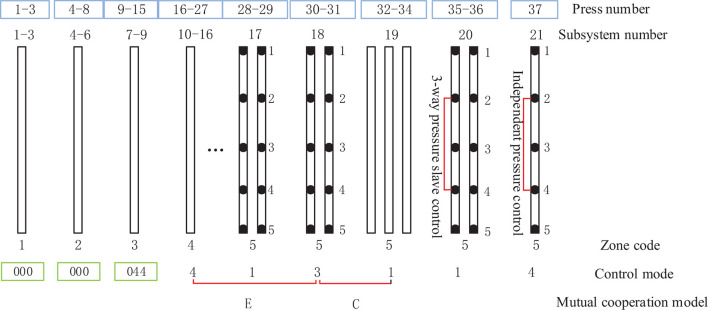
The stage zone division of frame groups of CPS cylinder array.

Based on the abovementioned distributed structure of the dynamic actuating unit in the cylinder array, the continuous flat press control mode sequence parameter was determined according to the dynamic information of the deviation type. For craft quality control, the control mode of the MDF continuous flat pressing machine was analyzed, and the coordinated control mode sequence between the continuous flat press frame cellular groups in the deviation correction process was programmed by the framework elements of the five abovementioned mutual cooperation modes, and the digital twin mapping was implemented between digital twin technology and deviation types, as shown in Figure 12.

The 21 subsystems comprising several frames of CPS were divided into five zones according to the self-defined hot-pressing craft stage, as shown in Figure 12. The subsystems 1–3, the control mode of which is mode 0, were divided into zone 1; subsystems 4–6 comprising frames 4–8, the control mode of which is mode 0 were divided into zone 2; the subsystems 7–9 comprising frames 9–15, the control modes of which are modes 0 and 4 were divided into zone 3; the subsystems 10–16 comprising frames 16–27, the control mode of which is mode 4 were divided into zone 4; and the subsystems 17–21 comprising frames 28–37, the control mode of which is sequence 1, 3, 1, 1, and 4 are divided into zone 5.

**Figure 13:**
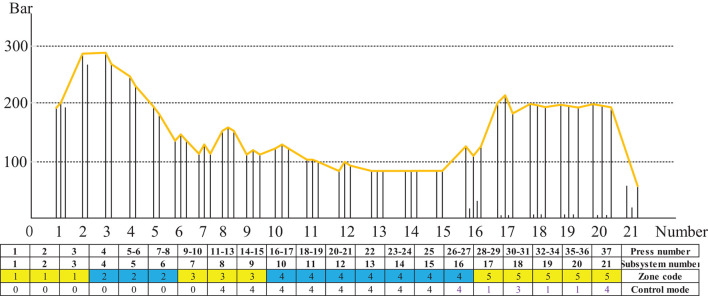
Sequence parameters programming of distributed control model for MDF continuous flat pressing process.

Figure 13 illustrates the three different zones of the continuous flat pressing pressure craft curve. In the rapid closing stage comprising subsystems 1–6, the pressure change setting must satisfy the requirements of the complete panel shape and the preset thickness after pre-pressing, generally showing the first half-sine trend. The subsystems 17–21 constitute the re-pressurization, thickness setting, and forming zone. In the pressure holding stage comprising subsystems 6–16, a certain pressure must be maintained so that the panel thickness meets the standard before entering the thickness forming zone. The control sequences arranged by the cooperative modes of the frame subsystems were programmed according to the abovementioned craft requirements.

The pressure craft curve in Figure 12 was tracked according to the pressure of continuous flat pressing in actual production. In the case of deviation, the control sequence of 21 subsystems was adjusted correspondingly. Using the abovementioned control mode and mutual cooperative method to analyze the effect of different deviation types in the thickness forming stage, the dynamic control sequence was programmed by real-time monitoring data, digital twins, and identifying the deviation types, as shown in Table 5. The programming was written in accordance with the on-site craft specifications and expert rules. In Table 5, *S* represents the sensor; the first digit of subscript numbers 10, 20, 21, 43, etc. represents the position number of the sensor; the second digit represents the thickness deviation level; and the five sensor symbol sequence parameters correspond to five types of deviations. Finally, the number sequences, such as 4-4-2-3-4, are the corresponding deviation elimination control strategies.

**Table 5:** Craft control sequence parameters programming for deviation types in the thickness forming stage.

**Sensor**	**Type of deviation**	**Control sequence parameters programming**
*S*_10_ *S*_20_ *S*_30_ *S*_40_ *S*_50_	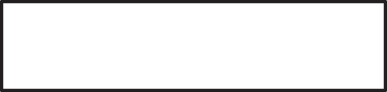	4-4-2-3-4
*S*_10_ *S*_21_ *S*_32_ *S*_43_ *S*_54_	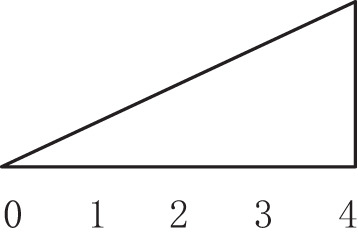	0-2-3-1-4
*S*_14_ *S*_23_ *S*_32_ *S*_41_ *S*_50_	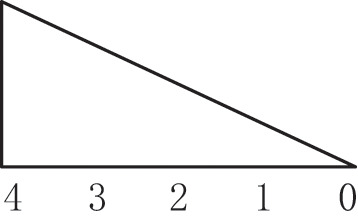	4-3-2-2-4
*S*_12_ *S*_21_ *S*_30_ *S*_41_ *S*_52_	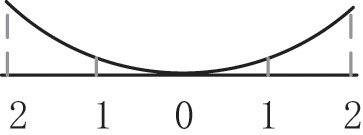	4-1-3-1-4
*S*_10_ *S*_21_ *S*_32_ *S*_41_ *S*_50_	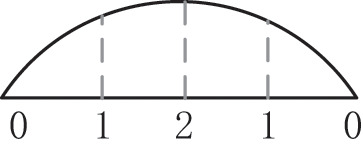	1-3-2-2-4

## 4. Application Testsed

Taking the CPS cylinder arrays of 37 frames on the MDF production line with an annual output capacity of 3,00,000 *m*^3^ as the research object, and product specifications of a 5.5 mm slab as an example, the distributed control mode framework and quality control collaborative programming method were tested and analyzed. The meanings of the arrows in the figure involved in the application test are that the red arrow “↑” means the pressure value increases and the green arrow “↓” reduces the pressure value.

**Figure 14:**
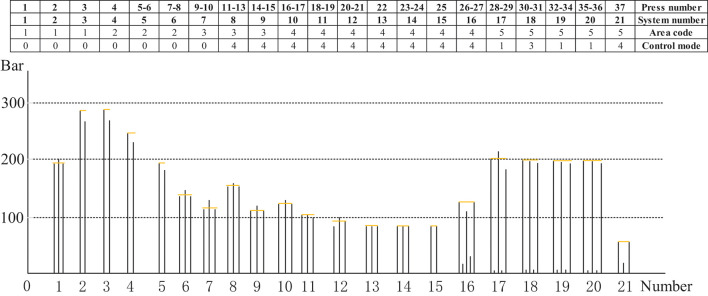
The frame subsystems pressure trend legend for production of 5.5 mm MDF.

The frame subsystem pressure trend legend for the production of 5.5 mm MDF is presented in Figure 14. Considering that the control modes adopted by the 21 subsystems of pressure cylinders are not identical, the specific control mode sequence is defined. For instance, subsystems 1–7 adopt control mode 0 by independent three-way control; subsystems 8–16 adopt mode 4 by pressure independent control, and subsystems 17–21 adopt control mode sequence parameter 1-3-1-1-4 by the leading–follower scheme.

**Figure 15:**
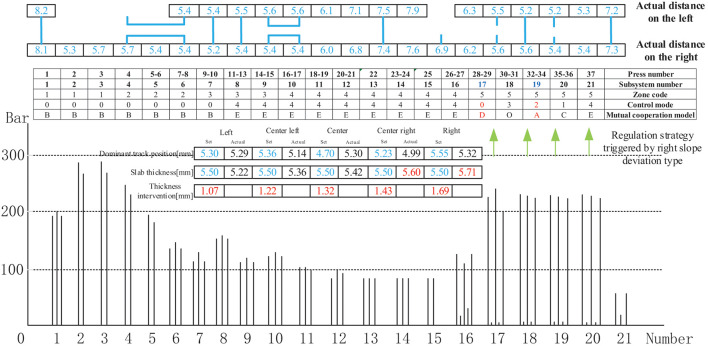
Control mode sequence regulation strategy triggered by right slope deviation type.

Figure 15 shows the control mode regulation scheme and pressure change triggered by the right-slope deviation type in the thickness forming stage. The actual distance between the left and right thickness intervention values, the intervention amount is based on the previous experience of experts. The given manual intervention value, the difference between the deviation value of each deviation type and the standard intervention amount range, the difference between different types of deviations is different, are generally based on the actual data on site. Which is used to guide the thickness deviation correction control mode scheduling, was within the controllable range of the craft. Moreover, the regulation result shows that the actual distance between the left and the right is relatively small. The adjustment method streamlines the subsystems 17,19 to control mode 0,2, respectively; converts subsystems 19 and 20 into mutual cooperation mode A; and cooperates with subsystem 18 to form mutual cooperation modes B and C to realize the entire distance control thickness measurement linkage.

**Figure 16:**
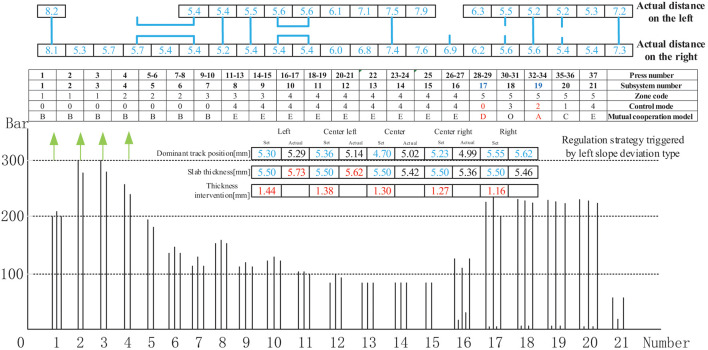
Control mode sequence regulation strategy triggered by left slope deviation type.

Figure 16 shows the control mode regulation scheme and pressure change triggered by the left-slope deviation type in the thickness forming stage. The adjustment method is identical to that described in Figure 13, except for the different intervention values.

**Figure 17:**
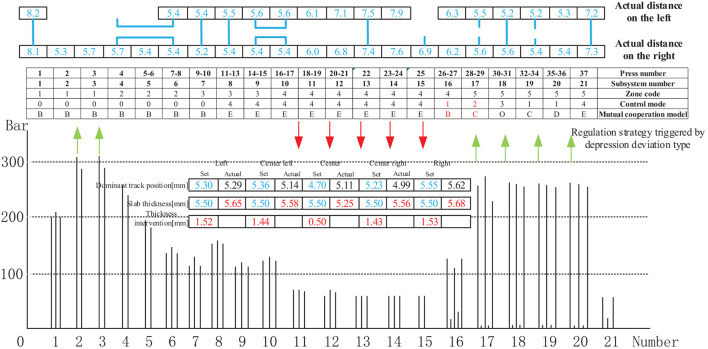
Control mode sequence regulation strategy triggered by depression deviation type.

Figure 17 shows the control mode regulation scheme and pressure change triggered by the depression deviation type in the thickness forming stage. From the pressure change diagram between the cellular cylinders in the case of depression, it can be observed that a depression phenomenon exists in the middle of the panel due to the excessive pressure of subsystems 11–15. Therefore, when adjusting the pressure, reducing the pressure value of subsystems 11–15 will minimize the possibility of depression in the middle. The actual distance between the depression deviation and the thickness intervention value is within the controllable range of the craft. Furthermore, the regulation result shows that the actual distance between the two sides and the middle of the panel is relatively small. The adjustment method streamlines the subsystems 16, 17 to control modes 1, 2, respectively and cooperates with subsystem 18 to form mutual cooperation modes B and C to realize the entire distance control thickness measurement linkage. Subsystem 20 adopts the cooperative mode of mutual cooperation mode D; the pressure is increased in subsystems 2–3 and 17–20, and subsystems 11–15 are operated at reduced pressure to achieve a slab thickness equal to the set value of 5.5 mm.

**Figure 18:**
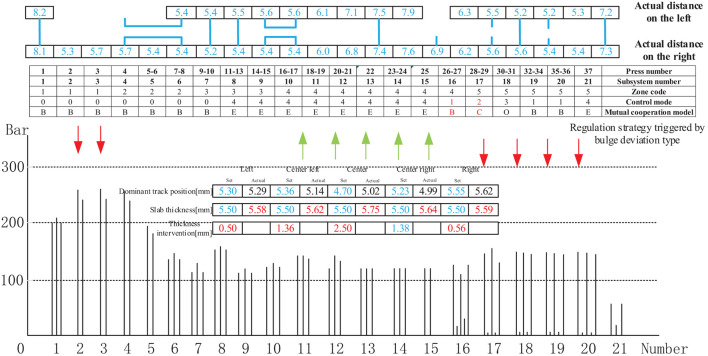
Control mode sequence regulation strategy triggered by bulge deviation type.

Figure 18 shows the control mode regulation scheme and pressure change triggered by the depression deviation type in the thickness forming stage. The adjustment method is identical to that described in Figure 15, except for the different intervention values.

The above application test demonstrates that various indicators such as thickness deviation, panel size deviation, surface quality, physical and mechanical property indicators, and other inspection indicators meet the national standards. In addition, the distributed control mode framework constructed by analyzing the deviation types of MDF continuous flat pressing panels provides a more effective method to realize the coordination function between the continuous flat pressing cylinder cellular frames. Furthermore, the dynamic sequence parameter control programming scheme has a positive effect on the precise forming deviation quality control and achieving the effect of precise slab forming. In conclusion, the test results confirmed the feasibility and effectiveness of the proposed method implementation scheme.

## Conclusion

Using the CPS cylinder arrays of 37 frames as the research object, and focusing on the quality control of panel deviation, a framework of five distributed flat pressing cooperative control modes was constructed. In addition, a mutual coordination mechanism for the control mode was established between cylinder groups based on the master-slave structure. Furthermore, the forming control sequences of continuous flat pressing were programmed according to the production craft and quality control requirements, and a corresponding control scheme was provided. The proposed method was applied and tested on an MDF production line with an annual output capacity of 3,00,000 *m*^3^, which significantly improved the fiberboard premium product rate up to 95%. It is convenient for realizing deviation type identification and diagnosis used by real-time detection data, improves the adjustment response speed, and effectively solves the problem of precise slab forming control of thickness, slope, depression, bulge, etc. quality deviation caused in actual production.

However, a few limitations remain. For instance, the control programming problems between different levels in the deviation types, which is the precise regulation sequence of each deviation type in terms of specific classification level and intensity, need to be further researched and expanded.

## Data Availability Statement

The original contributions presented in the study are included in the article/supplementary material, further inquiries can be directed to the corresponding author/s.

## Author Contributions

YUL and YAL proposed the coordination mode framework and quality control collaborative programming method. YAL provided the experimental test platform field and provided information about research reports and results of the primary sources of the hot-pressing mechanism model from the National Natural Science Foundation of China. WJ conceived and guided the review and editing of the entire thesis. All authors have read and agreed to the published version of the manuscript.

## Funding

This research was funded by Cultivating Excellent Doctoral Dissertation of Forestry Engineering No. LYGCYB202008, the Fundamental Research Funds for the Central Universities No. 2572021AW08, and also funded by Nation National Natural Science Foundation Grant of China No. 31370565.

## Conflict of Interest

The authors declare that the research was conducted in the absence of any commercial or financial relationships that could be construed as a potential conflict of interest.

## Publisher's Note

All claims expressed in this article are solely those of the authors and do not necessarily represent those of their affiliated organizations, or those of the publisher, the editors and the reviewers. Any product that may be evaluated in this article, or claim that may be made by its manufacturer, is not guaranteed or endorsed by the publisher.
